# Supplementary material for: Rediscovering the Rete Ovarii: a secreting auxiliary structure to the ovary
Source: bioRxiv. 2024 Nov 11:2023.11.08.566085. Originally published 2023 Nov 11. Preprint. [Version 3] doi: 10.1101/2023.11.08.566085 (PMC10659334; doi:10.1101/2023.11.08.566085)
Supplement: Supplement 2 [file media-2.pdf]

Rediscovering the *Rete Ovarii*: a secreting auxiliary structure to the ovary

Dilara N. Anbarci[1], Jennifer McKey1[1,2], Daniel S. Levic[1], Michel Bagnat[1], and Blanche Capel[1]*

SUPPLEMENTAL INFORMATION

Supplemental Figure 1

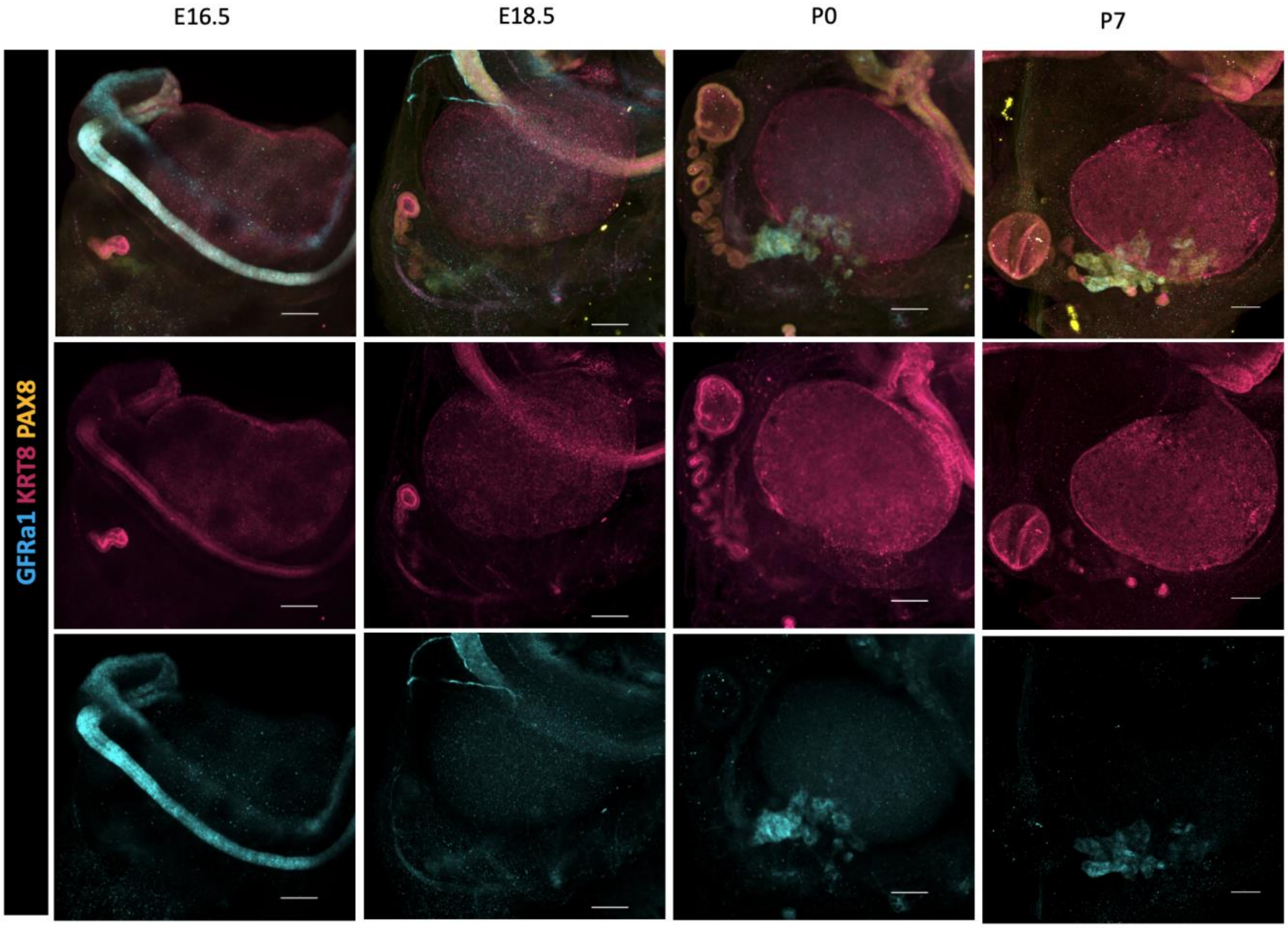

Supplemental Figure 1. Ventral view of ovary and EOR during development.

*Maximum intensity projection from confocal Z-stacks of whole ovary/mesonephros complexes at E16.5 (a), E18.5 (b), P0 (c) and P7 (d) immunostained for PAX8 (yellow), GFRa1 (cyan) and KRT8 (magenta). Top row are composite images, while the third row show separate panels for KRT8 and and the bottom row show panels for GFRa1. *Note that GFRa1 and KRT8 do not co-localize and are specific to the CR and EOR, respectively. All figures are dorsal views of the ovary. Yellow asterisk indicates opening of the infundibulum for reference. Scale bar – 100um*

Supplemental Table 1

Gene Name	Sequence 5' to 3'
mTmG (Mutant Reverse)	AAT CCA TCT TGT TCA ATG GCC GAT C
mTmG (Wildtype Forward)	GGC TTA AAG GCT AAC CTG ATG TG
mTmG (Mutant Forward)	CCG GAT TGA TGG TAG TGG TC
mTmG (Wildtype Reverse)	GGA GCG GGA GAA ATG GAT ATG
Tre-Cre (Forward)	GCG GTC TGG CAG TAA AAA CTA TC
Tre-Cre (Reverse)	GTG AAA CAG CAT TGC TGT CAC TT
Pax8-rtTa (Forward)	CCA TGT CTA GAC TGG ACA AGA
Pax8-rtTa (Reverse)	CTC CAG GCC ACA TAT GAT TAG
Tre-H2B-GFP (Forward)	GCT CGT TTA GTG AAC CGT CAG
Tre-H2B-GFP (Reverse)	TCT TCT GCG CCT TAG TCA CC
LGR5 (Mutant Forward)	CTG AAC TTG TGG CCG TTT AC
LGR5 (Wildtype Reverse)	GTC TGG TCA GAA TGC CCT TG
LGR5 (Common)	CTG CTC TCT GCT CCC AGT CT

Supplemental Table 1. Table recapitulating the DNA forward and reverse primers used for genotyping the transgenic mouse lines used in the present study

Supplemental movie legend

Supplemental Movie 1. 3D model of an XX ovarian complex at E18.5

This video depicts the 3D rendering of native lightsheet images of an E18.5 ovary/mesonephros complex. Demonstrating how the rete wraps around the ovary. DNA (grey) Ovarian Surface epithelium (green) PAX8 (red).
